# Results of heart transplantation: 18 years experience in University Hospital Dubrava, Zagreb

**DOI:** 10.1186/1749-8090-8-S1-O151

**Published:** 2013-09-11

**Authors:** M Planinc, Ž Sutlić, I Rudež, D Barić, D Unić, R Blažeković, M Grubišić, J Varvodić, B Starčević, D Planinc

**Affiliations:** 1 Department of Cardiac and Transplant Surgery, University Hospital Dubrava, Zagreb, Croatia; 2 Department of Cardiovascular Diseases, University Hospital Dubrava, Zagreb, Croatia; 3 Department of Cardiology, University Clinical Center Sisters of Mercy, Zagreb, Croatia

## Background

Despite advances in medical treatment, development of new surgical procedures and the availability of mechanical circulatory support heart transplantation (HTx) remains the treatment of choice for end-stage heart failure. Objective of this study is to report single center experience and outcomes of patients undergoing HTx.

## Methods

We retrospectively examined the outcomes from ninety-five HTx recipients between September 1995 and May 2013. The mean recipient age was 55±8 years, and 85% were male. Dilated cardiomyopathy was present in 40%, ischemic in 30% and 30% were other causes. Ten patients (10%) that were heart recipients from our cohort were on high urgent list of Eurotransplant. As an induction of immunosuppressive therapy we were using antithymocyte globulin, and for maintenance a combination of cyclosporine, prednisone and mycophenolate. Survival was studied using Kaplan-Meier curves.

## Results

In-hospital mortality was 12%. The median follow-up was 20 months. The global survival rates at 1, 5, and 10 years were 82%, 77%, and 62% respectively. The mean survival is 105 months (95% CI, 93.4-118.3). Early main causes of death were sepsis (41%) and primary graft failure (29%) and late causes were late rejection (20%), malignant disease and other causes (10%).

## Conclusion

In our center, post-HTx survival rates at 1, 5, and 10 years were even better than those reported by the International Society of Heart and Lung Transplantation as a result of combined effort of all medical personnel involved in perioperative and postoperative management.

